# Direct and indirect spino-cerebellar pathways: shared ideas but different functions in motor control

**DOI:** 10.3389/fncom.2015.00075

**Published:** 2015-07-06

**Authors:** Juan Jiang, Eiman Azim, Carl-Fredrik Ekerot, Bror Alstermark

**Affiliations:** ^1^Department of Integrative Medical Biology, Section of Physiology, Umeå UniversityUmeå, Sweden; ^2^Departments of Neuroscience and Biochemistry and Molecular Biophysics, Howard Hughes Medical Institute, Kavli Institute for Brain Science, Mortimer B. Zuckerman Mind Brain Behavior Institute, Columbia UniversityNew York, NY, USA; ^3^Department of Experimental Medical Science, University of LundLund, Sweden

**Keywords:** lateral reticular nucleus (LRN), spino-cerebellar pathways, spino-LRN-cerebellar pathways, internal feedback, motor control

## Abstract

The impressive precision of mammalian limb movements relies on internal feedback pathways that convey information about ongoing motor output to cerebellar circuits. The spino-cerebellar tracts (SCT) in the cervical, thoracic and lumbar spinal cord have long been considered canonical neural substrates for the conveyance of internal feedback signals. Here we consider the distinct features of an indirect spino-cerebellar route, via the brainstem lateral reticular nucleus (LRN), and the implications of this pre-cerebellar “detour” for the execution and evolution of limb motor control. Both direct and indirect spino-cerebellar pathways signal spinal interneuronal activity to the cerebellum during movements, but evidence suggests that direct SCT neurons are mainly modulated by rhythmic activity, whereas the LRN also receives information from systems active during postural adjustment, reaching and grasping. Thus, while direct and indirect spino-cerebellar circuits can both be regarded as internal copy pathways, it seems likely that the direct system is principally dedicated to rhythmic motor acts like locomotion, while the indirect system also provides a means of pre-cerebellar integration relevant to the execution and coordination of dexterous limb movements.

## Introduction

**Figure 1:**
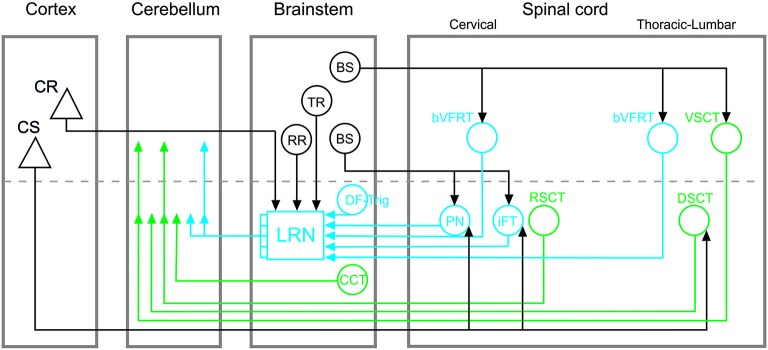
**Overview of direct spino-cerebellar and indirect spino-LRN-cerebellar mossy fiber pathways**. Direct spino-cerebellar pathways are indicated in green: the ventral spino-cerebellar tract (VSCT) and dorsal spino-cerebellar tract (DSCT) originate in thoracic and lumbar segments; the rostral spino-cerebellar tract (RSCT) originates in cervical segments; the cuneo-cerebellar tract (CCT) originates in the brainstem. Indirect spino-LRN-cerebellar pathways are indicated in blue: the bilateral ventral flexor reflex tract (bVFRT) originates in cervical and lumbar segments; the ipsilateral forelimb tract (iFT) and propriospinal neurons (PN) originate in cervical segments; and the dorsal funiculus-trigeminal tract (DF-Trig) originates in the brainstem. Green and blue arrowheads in the cerebellum indicate ipsi- and contralateral terminations (on either side of the dashed line). In the lateral reticular nucleus (LRN), discrete and convergent pathways convey information from the various spinal systems to the cerebellar cortex, though in this simplified circuit diagram they are illustrated by a combined mossy fiber output from the LRN. Descending inputs onto brainstem and spinal circuits are marked by black lines and arrowheads: cortico-spinal (CS), cortico-reticular (CR), rubro-reticular (RR), tecto-reticular (TR), and bulbo-spinal (BS). CS projections are to PN, iFT, and DSCT, but may consist of separate subpopulations. The CR, RR, and TR projections are to the LRN. The BS projections include different subpopulations: to bVFRT and VSCT mainly via the lateral vestibulo-spinal tract; to PN via the rubro-spinal, reticulo-spinal and tecto-spinal tracts; to iFT via the rubro-spinal tract.

Cerebellar circuits are of major importance in the control of movements, providing a neural basis for pattern recognition and motor behavioral correction and adaptation (Ito, 2006). These contributions to motor control depend on specific mossy fiber and climbing fiber cerebellar inputs that convey information about both ongoing motor output and external sensory events (Ito, 1984; Dean et al., 2010). In this paper, we focus on the organization of mossy fiber systems and, more specifically, we delineate two classes of spino-cerebellar pathways: direct spino-cerebellar projections, and indirect pathways via the lateral reticular nucleus (LRN; referred to as the spino-LRN-cerebellar pathway), as illustrated schematically in Figure 1.

Spino-cerebellar pathways have been implicated in the transmission of information about external events from various sensory modalities (cf. review, Stecina et al., 2013), the cancellation of reafferent sensory signals during self-generated movements (Hantman and Jessell, 2010), and the conveyance of internal copies of motor commands for rapid motor prediction and correction (Lundberg, 1971; Arshavsky et al., 1972, 1978; Alstermark and Isa, 2012; Fedirchuk et al., 2013; Azim and Alstermark, 2015). However, little is known about the organizational and functional logic underlying the existence of two separate systems for conveying spinal signals to the cerebellum. By comparing the phylogeny, anatomy, genetic identities and functional organization of direct and indirect spino-cerebellar circuits, we highlight key similarities and differences between these pathways and discuss principal questions that remain.

## Phylogeny

A phylogenetic comparison of cerebellar circuits has been extensively reviewed by Ito (1984). There is evidence that direct spino-cerebellar and indirect spino-LRN-cerebellar tracts coexist in teleost fish (Szabo et al., 1990; Finger, 2000), suggesting an early evolutionary divergence of these pathways. In mammals, several direct spino-cerebellar tracts (SCT) have been identified anatomically and electrophysiologically. Two of the most studied are the dorsal (DSCT) and ventral (VSCT) spino-cerebellar tracts that originate in the thoracic and lumbar spinal cord (Jankowska et al., 2011; cf. review: Stecina et al., 2013). The corresponding direct SCT for forelimb regions are the cuneo-cerebellar tract (CCT; Jansen and Brodal, 1954; Ekerot and Larson, 1972) in the brainstem and the rostral spino-cerebellar tract (RSCT; Oscarsson, 1965; Hirai et al., 1976) in cervical segments, respectively (Figure 1).

Indirect spino-LRN-cerebellar pathways have mainly been studied in the cat (Clendenin et al., 1974a,b,c,d, 1975; Matsushita and Ikeda, 1976; Ekerot, 1990a,b,c), but comparative anatomy (Walberg, 1952) has revealed the existence of the LRN in a large number of mammals including *Erinaceomorpha* (hedgehog), *Chiroptera* (bat), *Rodentia* (squirrel, mouse and rat), *Lagomorpha* (hare), *Carnivora* (cat, dog and seal), *Cetartiodactyla* (harbor porpoise), *Artiodactyla* (pig, cow and roe deer) and *Primates* (rhesus macaque and human).

Interestingly, in teleosts there are abundant axon collaterals from direct spino-cerebellar pathways to the LRN (Szabo et al., 1990), whereas in cats the DSCT does not provide collateral excitation to LRN neurons (Ekerot and Oscarsson, 1975). These phylogenetic differences suggest that direct spino-cerebellar and indirect spino-LRN-cerebellar pathways may have originated as cooperative systems, which became progressively separated as more advanced motor repertories evolved.

## Anatomy

As shown in Figure 1, direct spino-cerebellar and indirect spino-LRN-cerebellar pathways originate in cervical, thoracic and lumbar spinal segments, as well as in the brainstem (for review, cf. Alstermark and Ekerot, 2013; Pivetta et al., 2014). Within the direct and indirect classes, subpopulations with ipsilateral, contralateral and bilateral projections have been identified (cf. reviews: Alstermark and Ekerot, 2013; Stecina et al., 2013). The ultimate mossy fiber terminations of these pathways in the cerebellar cortex are found mainly in the vermal and paravermal regions of the anterior and posterior lobes, as well as in the paramedian lobe. The location of ascending axonal projections in the white matter of the spinal cord and the pattern of mossy fiber termination zones within the cerebellar cortex differ across individual systems, but broad comparison of direct and indirect pathways to each other reveals no clear differences (cf. review Ito, 1984). Thus, at least at the gross anatomical level, direct spino-cerebellar and indirect spino-LRN-cerebellar pathways target overlapping cerebellar cortical circuits.

## Genetic Identities

The genetic delineation of neuronal subtypes has complemented classical anatomical and electrophysiological characterization of spinal circuits, and has provided a means for selective manipulation and functional dissection of these pathways (Goulding, 2009). While the molecular identities of each of the direct spino-cerebellar systems are yet to be fully defined, studies in mice have revealed that a population of dorsally-derived spinal interneurons that express the transcription factor *Math1* give rise to multiple spino-cerebellar pathways (Bermingham et al., 2001). Moreover, DSCT neurons in Clarke’s column have been shown to selectively express the neurotrophic factor *Gdnf* (Hantman and Jessell, 2010).

Indirect spino-LRN-cerebellar pathways, and the cervical propriospinal neuron (PN) system in particular, have been the subject of much recent genetic scrutiny. A prominent population of excitatory PNs involved in goal-directed reaching movements was identified within the *Chx10*-expressing V2a interneuron class (Azim et al., 2014); notably, only cervical but not lumbar V2a interneurons project to the LRN, indicating that indirect LRN-cerebellar pathways originating in the lumbar cord have distinct genetic identities. In zebrafish, a subset of V2a spinal interneurons send ascending projections to the hindbrain (Menelaou et al., 2014), suggesting that the V2a interneuron class establishes an evolutionarily conserved circuit for the conveyance of motor signals to supraspinal regions. Moreover, recent genetic and viral labeling studies in mice have revealed that in addition to V2a interneurons, several classes of molecularly defined excitatory and inhibitory cervical spinal interneurons project to the LRN (Pivetta et al., 2014), suggesting that other indirect spino-cerebellar pathways can be dissected genetically along similar lines.

## Functional Organization

It has been well documented that both direct spino-cerebellar and indirect spino-LRN-cerebellar pathways convey information related to ongoing rhythmic movements, including locomotion, scratching and respiration (cf. references in reviews by Ito, 1984; Alstermark and Ekerot, 2013; Stecina et al., 2013). Moreover, it has been proposed that the VSCT (Lundberg and Weight, 1971) and DSCT (Hantman and Jessell, 2010) monitor the excitability of spinal interneurons. Interestingly, whereas the VSCT (Fedirchuk et al., 2013) and DSCT (Stecina et al., 2013) signal mainly during the flexion phase, spino-LRN-cerebellar pathways are active throughout the entire cycle of flexion and extension (cf review by Alstermark and Ekerot, 2013), suggesting that indirect pathways convey a broader range of motor signals.

Another major difference in the functional organization of direct and indirect cerebellar pathways is that the four subsystems in the indirect spino-LRN-cerebellar pathway originating in the cervical spinal cord and brainstem (Figure 1) may be dedicated to more than just rhythmic movements (Alstermark and Ekerot, 2013). These subsystems, by monitoring the excitability of spinal interneurons, could signal information about posture (bilateral ventral flexor reflex tract; bVFRT), reaching (C3-C4 propriospinal system; PN), grasping (ipsilateral forelimb tract; iFT) and jaw opening (dorsal funiculus-trigeminal tract; DF-Trig), and their convergence in the LRN might enable the coordination of these separate motor actions into coherent and smooth movements (Alstermark and Ekerot, 2013, 2015).

Among these indirect systems, the function of C3-C4 PNs has been investigated extensively in the cat, monkey, human and recently in the mouse (Alstermark and Isa, 2012; Azim et al., 2014). These studies have shown that PNs mediate motor commands for reaching by directly modulating the activity of forelimb-innervating motor neurons, while also conveying copies of these motor commands, via axon collaterals, to the LRN. Genetic manipulation of PNs in the mouse has revealed that this internal copy pathway recruits a cerebellar-motor feedback loop, providing a plausible neural substrate for the rapid updating and correction of ongoing forelimb motor output (Azim et al., 2014; Azim and Alstermark, 2015). The current lack of selective genetic access to other spino-LRN-cerebellar pathways has precluded similar exploration of their behavioral functions, yet evidence suggests that the cervical bVFRT, iFT and PN systems provide both discrete and convergent internal feedback signals to LRN-cerebellar circuits (Alstermark and Ekerot, 2013; Pivetta et al., 2014; Huma and Maxwell, 2015), potentially enabling the coordination of forelimb and postural motor control. A companion article discusses the extensive convergence of projections from these distinct systems in the LRN, providing a pre-cerebellar center for the integration of spinal signals and their modulation by descending motor cortical pathways (Alstermark and Ekerot, 2015).

## Open Questions and Future Directions

1. How do descending motor pathways modulate the direct and indirect spino-cerebellar tracts? Thus far, only the descending inputs to the cervical PN system have been investigated systematically (Alstermark and Lundberg, 1992; Alstermark and Isa, 2012; Azim et al., 2014). The convergence of descending pathways onto cervical PNs suggests a role for these neurons in integrating motor command signals and conveying copies of this information to LRN-cerebellar circuits. A better understanding of the descending inputs onto other direct and indirect cerebellar pathways should help to clarify their potential contributions to voluntary movements.
2. Which of the SCT convey internal copies of last-order interneuronal signals to motor neurons? A bifurcating pre-motor/internal copy pathway has been demonstrated for the PN system in the cat, monkey, human and mouse (Alstermark and Isa, 2012; Azim et al., 2014), and recent anatomical evidence in the mouse suggests that iFT and bVFRT systems might also send bifurcating projections directly to forelimb motor neurons and to the LRN (Pivetta et al., 2014). However, innervation of motor neurons by these pathways remains largely untested in other mammals. Studies in the cat suggest that bVFRT neurons do not project directly to lumbar motor neurons (Alstermark, Lundberg and Sybirska, unpublished findings), though direct projections to cervical motor neurons have not been explored.
3. Do any of the mammalian direct SCT send collaterals to the LRN, as they do in teleost? Studies of the DSCT suggest that collaterals to the LRN do not exist in the cat (Ekerot and Oscarsson, 1975), though additional anatomical and electrophysiological examination is needed to resolve whether the strict separation of direct and indirect cerebellar pathways is a distinguishing feature of mammalian motor circuits.
4. What is the function of the cortico-reticular (CR) projection to the LRN? This pathway may exert a modulatory top-down influence over the information conveyed from the spinal cord to the cerebellum. Genetic dissection of LRN neurons and their input pathways could help resolve the organization and function of descending control of LRN output by the cerebral cortex.
5. What are the behavioral contributions of each of the direct and indirect spino-cerebellar systems? The diversity of direct and indirect cerebellar pathways in the cervical cord in particular suggests that these systems may have evolved in concert with the increasing complexity of dexterous forelimb movements. The identification of unique genetic markers for each of these pathways should offer a means to access and manipulate these circuits selectively, providing the experimental resolution needed to characterize their discrete contributions to motor behavior (Azim et al., 2014; Azim and Alstermark, 2015).
6. There is growing interest in applying computational neurobiology approaches to understanding the molecular and genetic mechanisms that may contribute to spino-cerebellar ataxia (cf. review by Brown et al., 2015), and models devoted to the role of internal feedback more generally have explored various neural circuits in the cortex, brainstem and spinal cord (cf. review by Azim and Alstermark, 2015). Regarding spinocerebellar pathways, a hypothesis has been forwarded on their role in the multi-dimensional integration of sensorimotor information (Spanne and Jörntell, 2013). However, in this model, direct spino-cerebellar and indirect spino-LRN-cerebellar pathways are grouped together. A new hypothesis has recently been proposed that focuses specifically on the role of indirect spino-LRN-cerebellar pathways (Alstermark and Ekerot, 2013). Future modeling approaches, informed by the experimental work described above, should provide greater insight into the discrete functions of direct and indirect spino-cerebellar systems.

## Conflict of Interest Statement

The authors declare that the research was conducted in the absence of any commercial or financial relationships that could be construed as a potential conflict of interest.
